# Patient-reported pain severity is associated with shorter survival in older adults with newly diagnosed cancer

**DOI:** 10.1007/s00520-025-09779-x

**Published:** 2025-07-26

**Authors:** Jennifer Gabbard, Scott Isom, Tiffany Statler, Joanna Asselin, Kathryn E. Callahan, Nicholas M. Pajewski, Lynne I. Wagner, Armida Parala-Metz, Janet A. Tooze, Heidi D. Klepin

**Affiliations:** 1 Department of Internal Medicine, Section of Gerontology and Geriatrics, School of Medicine, Wake Forest University, 1 Medical Center Boulevard, Winston-Salem, NC 27157 USA; 2 Department of Biostatistics and Data Science, Wake Forest University School of Medicine, Winston-Salem, NC USA; 3 Department of Social Sciences & Health Policy, School of Medicine, Wake Forest University, Winston-Salem, NC USA; 4 Department of Hematology and Oncology, Atrium Health, Charlotte, NC USA; 5 Department of Cancer Medicine, Wake Forest University School of Medicine, Winston-Salem, NC USA

**Keywords:** Patient-reported outcome measures, Pain, Cancer, Frailty, Survival

## Abstract

**Purpose:**

Pain is a prevalent and often undertreated symptom among older adults with cancer. This study examined whether self-reported pain levels are associated with overall survival (OS) in older adults newly diagnosed with cancer receiving chemotherapy.

**Methods:**

This retrospective cohort study included patients aged ≥ 65 years with newly diagnosed lung, colorectal, or breast cancer between 2017 and 2020, identified via a cancer registry at an academic medical center. Self-reported pain using the numeric rating scale (0–10), documented within 30 days of chemotherapy initiation, was categorized as none (0), mild (1–4), moderate (5–7), or severe (8–10). Baseline demographics, opioid use, and frailty (measured using a deficit accumulation approach) were collected. Kaplan–Meier was used to compare OS between groups. Cox proportional hazards models evaluated the association between pain levels and OS, adjusting for demographics, cancer stage and type, opioid use, and frailty.

**Results:**

Among 509 patients (median age 72.2 years; 55% female; 83.5% white; 13.4% Black), pain was highly prevalent: 16.5% reported mild, 19.6% moderate, and 17.3% severe pain. Median OS was significantly lower for patients with severe pain (9.1 months, 95% CI 6.5–12.7) compared to those with no pain (29.1 months, p < 0.0001). Severe pain (HR 1.60, 95% CI 1.14–2.24) and moderate pain (HR 1.52, 95% CI 1.10–2.10) were independently associated with higher mortality.

**Conclusions:**

Self-reported pain was significantly associated with reduced OS in older adults with cancer. These findings suggest that self-reported pain may serve as a clinical indicator of poor prognosis and support the role of routine pain assessment in identifying high-risk patients to support patient-centered care.

**Supplementary Information:**

The online version contains supplementary material available at 10.1007/s00520-025-09779-x.

## Introduction

The burden of cancer disproportionately affects older adults, with approximately half of all new cancer diagnoses occurring in individuals aged 66 and older. A significant symptom experienced by this population is pain, a symptom reported by around 45% of patients undergoing cancer treatment and by 66% of those with advanced cancer [1]. Among these patients, appropriately 30% experience moderate or severe pain [2, 3]. Despite advancements made in pain management, pain in older adults with active cancer is often unrecognized and under-treated, contributing to impaired health-related quality of life, worsening function, and increased healthcare utilization [1].

Older adults are particularly vulnerable to the undertreatment of pain, in part due to hesitancy around prescribing medications in this population, concerns about increased sensitivity to side effects, polypharmacy, and age-related changes in drug metabolism that complicate pain management [4]. Tools for patient-reported pain assessment have proven reliable in screening for uncontrolled pain among cancer patients [5]. Although several studies have examined the role of pain in survival among specific cancer types, including head and neck, prostate, and breast cancer [6–14], there remains a critical gap in understanding the broader impact of pain on survival in older adults with cancer, particularly across common solid tumors like lung, breast, and colorectal cancer. Existing literature has largely focused on quality-of-life impacts, with limited exploration of pain as a potential risk factor for mortality. This study addresses that gap by examining the association between self-reported pain within 30 days of initiating chemotherapy and overall survival (OS) in older adults with newly diagnosed cancer (either lung, breast, or colorectal cancer).

Given the high prevalence and clinical relevance of frailty in this population, we also conducted an exploratory analysis to describe the relationship between frailty and pain [15–19]. While frailty was not a primary focus of this study, its frequent co-occurrence with pain warranted additional examination to characterize the broader vulnerability of this population [20–26]. These exploratory findings may offer additional insight into the symptom burden experienced by older adults with cancer and inform future studies aimed at improving risk stratification and supportive care.

## Methods

### Study design, population, and data sources

This study was a retrospective cohort analysis utilizing cancer registry data, supplemented by data from the electronic health record (EHR). The study was conducted at Atrium Health Wake Forest Baptist Medical Center, a large academic medical center and regional referral center serving a 20-county area in western North Carolina. Patients were included in the study if they were (1) aged ≥ 65 years, (2) newly diagnosed with lung, breast, or colorectal cancer between 2017–2020, (3) initiating chemotherapy, and (4) had a self-reported pain within 30 days of chemotherapy. This study was approved by the Atrium Health Wake Forest Baptist Institutional Review Board. Data pulled from the Cancer Registry included age at diagnosis, sex, race, ethnicity, cancer type, stage at diagnosis, date of first oncology visit, chemotherapy start dates, and date of death. Race and ethnicity were combined into a single variable using self-reported data, and categorized as non-Hispanic White, non-Hispanic Black, Hispanic, or Other. All analyses referring to this variable use the combined term “race and ethnicity.” Data pulled from the EMR included patient-reported measures, clinical data (e.g. medications), opioid use prior to starting chemotherapy, healthcare encounter data, and vital status (i.e. date of death or last known follow-up). Cancers were staged according to the American Joint Committee on Cancer (AJCC) 7th staging system. Overall survival was calculated from the date of diagnosis to the date of death (from either the EHR or the cancer registry; typically the EMR was more up to date) and was censored at the time of last follow-up. Additionally, the Area Deprivation Index (ADI) was calculated using patients’ residential addresses to provide a standardized measure of socioeconomic disadvantage at the neighborhood level.

### Measures

#### Pain assessment

The numeric rating scale (NRS) was used to assess pain severity on a scale of 0–10. On this scale, 0 indicates"no pain,"and 10 indicates"the worst pain imaginable."Pain scores were abstracted from the EHR if recorded within 30 days of initiating chemotherapy; in most cases, these were collected at or shortly after treatment initiation during routine outpatient visits. When multiple scores were available, the highest score closest to the chemotherapy start date was used in the analysis. Pain levels were categorized as none (0), mild (1–4), moderate (5–7), or severe (8–10).

#### Frailty

Frailty was assessed using the electronic frailty index (eFI) at the time of diagnosis. The construction of the eFI has been described previously [27, 28]. Briefly, the eFI quantifies the proportion of age-related deficits present for an individual based on the deficit accumulation model of frailty and integrates over 50 total deficits constructed from diagnosis codes, vital signs, laboratory measurements, smoking history, medications, and, if available, functional assessments drawn from Medicare annual wellness visits. Frailty status was categorized as fit (eFI ≤ 0.10), pre-frail (0.10 < eFI ≤ 0.21), frail (eFI > 0.21), or non-calculable. The electronic frailty index (eFI) is based on the proportion of age-related deficits present over the total number evaluated (score range 0–1) [27]. In this implementation, the eFI was only calculated for individuals with at least two outpatient visits that included blood pressure readings within the past two years. This threshold was chosen to ensure a minimal opportunity for diagnosis coding and clinical assessment; individuals without two qualifying visits were categorized as having a non-calculable frailty score.

### Analysis

Patient demographics were summarized using mean and standard deviation, median and interquartile range, or counts and percentages based on the distributions of the characteristics. The association of pain with overall survival was evaluated initially using Kaplan–Meier plots and a log-rank test, then using a Cox proportional hazards model adjusting for age, sex, race, cancer stage, cancer type, opioid use, and frailty status. The association between frailty and pain was investigated using a chi-square test. For categorical variables with more than two levels, we report the global (omnibus) p-value from the Wald chi-square test to assess the overall significance of the variable in the multivariable Cox model. Individual hazard ratios and confidence intervals are presented for each category relative to the reference group. All analysis was done with SAS version 9.4 (SAS Institute Inc. Cary, NC).

## Results

### Patient demographics and patient-reported pain levels

Table 1 displays the overall demographics of our study cohort and patient-reported pain levels. 509 adults were included in the cohort (median age 72.2 years, 55% female, 83.5% white, and 13.4% black) with lung (N = 312, 61.3%), colorectal (N = 111, 21.8%) and breast (N = 86, 16.9%) cancer. Nearly half of the patients were diagnosed with stage 4 (or metastatic) disease. In regard to frailty status, most were pre-frail (42.4%) followed by fit (27.7%), frail (14.9%) and not calculable (14.9%). Frailty was highest among those with lung cancer. In the overall sample, the distribution of self-reported pain categories (documented in 504 of the 509 patients, 99%) was mild (N = 84, 16.5%), moderate (N = 100, 19.6%), severe (N = 88, 17.3%), and none (N = 232, 45.6%); 37% reported moderate-severe pain with the highest uncontrolled pain levels reported among those with active lung cancer. Of the 509 patients in the cohort, 290 (57.0%) died during the follow-up period, while 219 (43.0%) were censored at their last known follow-up visit.

**Table 1:** Patient Demographics

*N* (%)	All *N* = 509	Breast *N* = 86	Lung *N* = 312	Colorectal *N* = 111
Age at chemo (years), median (IQR)	72.2 (68.6; 76.8)	70.9 (67.7; 74.1)	72.7 (69.2; 77.1)	71.5 (68.2; 77.2)
Sex				
Female	280 (55.0%)	85 (98.8%)	138 (44.2%)	57 (51.4%)
Male	229 (45.0%)	1 (1.2%)	174 (55.8%)	54 (48.6%)
Race/Ethnicity				
White	425 (83.5%)	68 (79.1%)	261 (83.7%)	96 (86.5%)
Black	68 (13.4%)	16 (18.6%)	42 (13.5%)	10 (9.0%)
Hispanic	5 (1.0%)	0 (0.0%)	3 (1.0%)	2 (1.8%)
Other	11 (2.2%)	2 (2.3%)	6 (1.9%)	3 (2.7%)
eFI				
Fit	141 (27.7%)	37 (43.0%)	69 (22.1%)	35 (31.5%)
Pre-Frail	216 (42.4%)	37 (43.0%)	136 (43.6%)	43 (38.7%)
Frail	76 (14.9%)	9 (10.5%)	51 (16.3%)	16 (14.4%)
Not Calculable	76 (14.9%)	3 (3.5%)	56 (17.9%)	17 (15.3%)
Stage				
1	53 (10.4%)	42 (48.8%)	9 (2.9%)	2 (1.8%)
2	57 (11.2%)	23 (26.7%)	25 (8.0%)	9 (8.1%)
3	145 (28.5%)	6 (7.0%)	98 (31.4%)	41 (36.9%)
4	233 (45.8%)	12 (14.0%)	170 (54.5%)	51 (45.9%)
Unknown	21 (4.1%)	3 (3.5%)	10 (3.2%)	8 (7.2%)
Area of Deprivation Index (Median, IQR)	70.0 (55.0; 82.0)	67.5 (53.0; 85.0)	71.0 (58.0; 82.0)	66.0 (49.5; 78.0)
Use of Opioids				
No	390 (76.6%)	57 (66.3%)	234 (75.0%)	99 (89.2%)
Yes	119 (23.4%)	29 (33.7%)	78 (25.0%)	12 (10.8%)
Pain				
None	232 (45.6%)	50 (58.1%)	124 (39.7%)	58 (52.3%)
Mild	84 (16.5%)	17 (19.8%)	50 (16.0%)	17 (15.3%)
Moderate	100 (19.6%)	15 (17.4%)	66 (21.2%)	19 (17.1%)
Severe	88 (17.3%)	3 (3.5%)	70 (22.4%)	15 (13.5%)
Missing	5 (1.0%)	1 (1.2%)	2 (0.6%)	2 (1.8%)

*eFI* electronic frailty index; *IQR* interquartile range

Frailty was assessed using the electronic frailty index (eFI) and categorized as fit (eFI ≤ 0.10), pre-frail (0.10 < eFI ≤ 0.21), frail (eFI > 0.21), or not calculable. eFI scores were only calculated for individuals with ≥ 2 outpatient visits including blood pressure measurements in the two years prior to cancer diagnosis. Race and ethnicity were combined into a single variable using self-reported data and categorized as White, Black, Hispanic, or Other

### Relationship between pain and survival

**Fig. 1:**
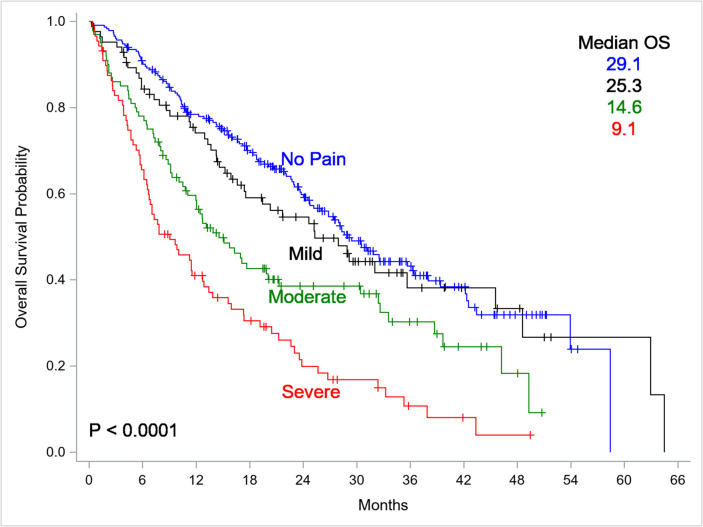
Kaplan-Meier Curves of Overall Survival by Self-Reported Pain Category Among Older Adults with Cancer Receiving Chemotherapy (*N* = 509). Kaplan–Meier survival curves demonstrating the relationship between self-reported pain levels (None, Mild, Moderate, Severe) within 30 days of initiating chemotherapy and overall survival (OS) among older adults (≥ 65 years) newly diagnosed with lung, breast, or colorectal cancer. Pain levels were categorized based on the Numeric Rating Scale (NRS): None (0), Mild (1–4), Moderate (5–7), and Severe (8–10). Median OS is displayed for each pain category, with severe pain associated with significantly lower survival (log-rank p < 0.0001)

Figure 1 shows the relationship between self-reported pain levels within 30 days of initiating chemotherapy and survival after initiating chemotherapy. Self-reported pain level was associated with survival (log-rank p < 0.0001) comparing the four pain levels. Median overall survival was 9.1 months (95% CI: 6.5, 12.7) for patients with severe pain, 14.5 (11.0, 20.1) months for those with moderate pain, 25.3 (17.4, 45.5) months for those with mild pain, and 29.1 (24.7, 36.3) months for those with no patient reported pain.

### Relationship between pain and mortality

Table 2 shows the relationship between patient-reported pain levels and mortality. After adjusting for age, sex, race, cancer stage, cancer type, opioid use, and frailty status, pain level was significantly associated with higher mortality among those with severe pain (Hazard Ratio (HR) 1.52, Confidence Interval (CI) 1.10–2.10) and moderate pain (HR 1.60, 95% CI 1.14–2.24) as compared to patients without pain. Notably, opioid use prior to chemotherapy was not independently associated with mortality (HR 0.87, 95% CI 0.65–1.17, p = 0.36). However, opioid use was more common among patients with higher pain levels—for example, 49% of those with severe pain had prior opioid use, compared to 12% of those reporting no pain—suggesting a positive correlation between pain severity and opioid use (Supplemental Table 1).

**Table 2:** Multivariable Association Between Patient-Reported Pain and Mortality Among Older Adults with Cancer Receiving Chemotherapy (N = 509)

	Hazard Ratio (95% Confidence Interval)	*p*-value
Pain (ref = No Pain)		**0.0099**
Mild	0.97 (0.68, 1.38)	
Moderate	1.52 (1.10, 2.10)	
Severe	1.60 (1.14, 2.24)	
Age (10-year increase)	1.84 (1.49,2.28)	** < 0.0001**
Male (ref = Female)	1.17 (0.92,1.49)	**0.2056**
Race/Ethnicity (ref = White)		**0.1391**
Black	0.96 (0.68,1.36)	
Hispanic	0.35 (0.09, 1.45)	
Other	2.16 (0.94, 4.98)	
Stage (ref = 1–3)		** < 0.0001**
4	2.91 (2.23, 3.78)	
Unknown	1.01 (0.52, 1.97)	
Cancer Type (ref = Breast)		** < 0.0001**
Lung	3.29 (1.94, 5.60)	
Colorectal	1.32 (0.73, 2.37)	
Opioid Use (ref = no)	0.87 (0.65, 1.17)	**0.3633**

Multivariable Cox proportional hazards model adjusted for all variables shown, including age, sex, race and ethnicity, cancer stage, cancer type, opioid use, and frailty status. p-values shown in the right column represent the overall significance of categorical variables with multiple levels (e.g., pain level, race/ethnicity, stage, cancer type). Hazard ratios are shown relative to the reference group listed in parentheses

### Relationship between frailty and pain

Table 3 displays the distribution of patient-reported pain levels across frailty categories, as measured by the electronic frailty index (eFI). Pain severity was significantly associated with frailty status (p < 0.0001), with higher pain levels observed among individuals classified as frail. Among those categorized as fit, the majority reported experiencing no pain (59.3%), while 20.0% reported mild pain, 13.6% reported moderate pain, and 7.1% reported severe pain. In the pre-frail group, a lower proportion reported no pain (49.5%) or mild pain (15.6%), with higher percentages experiencing moderate (18.4%) or severe pain (16.5%). Among individuals classified as frail, fewer reported no pain (30.3%), with higher frequencies of mild (19.7%), moderate (21.1%), and severe pain (29.0%). Notably, among individuals for whom frailty status could not be calculated, a substantial proportion reported moderate (34.2%) or severe pain (27.6%). While this analysis highlights the frequent co-occurrence of pain and frailty in this population, frailty was treated as a confounder in this study’s survival analysis rather than as a mediator or modifier of the pain-mortality relationship.

**Table 3:** Relationship Between Frailty Status and Self-Reported Pain Levels Among Older Adults with Cancer Receiving Chemotherapy (N = 509)

*N* (%)	No Pain	Mild Pain	Moderate Pain	Severe Pain	*P*-value
eFI					< 0.0001
Fit	83 (59.3%)	28 (20.0%)	19 (13.6%)	10 (7.1%)	
Pre-Frail	105 (49.5%)	33 (15.6%)	39 (18.4%)	35 (16.5%)	
Frail	23 (30.3%)	15 (19.7%)	16 (21.1%)	22 (29.0%)	
Not Calculable	21 (27.6%)	8 (10.5%)	26 (34.2%)	21 (27.6%)	

*eFI* electronic frailty index

Frailty was categorized using the eFI as fit (eFI ≤ 0.10), pre-frail (0.10 < eFI ≤ 0.21), frail (eFI > 0.21), or not calculable. eFI scores were not calculated for individuals without ≥ 2 outpatient visits including blood pressure measurements in the 2 years prior to diagnosis. *P*-value represents the association between frailty category and pain level using a chi-square test

## Discussion

This study demonstrates a significant association between patient-reported pain levels and overall survival in older adults newly diagnosed with cancer. Higher self-reported pain levels at the onset of chemotherapy were associated with significantly lower survival rates, even after adjusting for cancer type, cancer stage, frailty status, opioid use, and demographic factors. While pain is a well-recognized symptom in cancer, our findings add to the growing body of literature highlighting its prognostic significance in survival outcomes [6–14, 29, 30]. Pain may reflect unmeasured clinical deterioration not captured by cancer stage or frailty status and may also serve as a surrogate for other dimensions of disease severity, such as tumor-related complications or symptom burden not fully reflected in traditional staging systems. However, the independent association with mortality underscores its potential prognostic value. These results underscore the critical need for early pain assessment and intervention, suggesting that pain may serve as a potentially modifiable risk factor in clinical management. Improving pain control may not only enhance quality of life but also potentially improve survival outcomes.

The biological mechanisms underlying the association between pain and survival remain unclear. However, prior research suggests that chronic and uncontrolled pain may contribute to cancer progression through immunosuppressive effects and chronic inflammation [10, 31–33]. Additionally, pain is associated with physical inactivity, sleep disturbances, and psychological stress, all of which may contribute to physiological decline and reduced resilience [34–37]. These mechanisms may explain why higher pain levels are associated with decreased survival in this cohort. Further research is needed to elucidate the specific pathways—such as inflammatory and immune dysregulation—through which pain impacts survival, as this could inform targeted interventions to improve both symptom management and oncologic outcomes. In exploratory analyses, we also observed that frailty was associated with higher pain levels, consistent with prior studies showing that these conditions frequently coexist in older adults with cancer [38–40]. While frailty was treated as a confounder in our primary analyses, its potential role as a mediator or modifier warrants future investigation to better understand the interplay between pain, frailty, and survival, particularly given their potential bidirectional relationship and the possibility that frailty may mediate or modify the impact of pain on outcomes [41, 42].

Emerging evidence suggests that lifestyle factors—including physical inactivity, sleep disturbances, smoking, and diet—play a key role in modulating pain severity [43]. A large Survey of Health, Ageing and Retirement in Europe (SHARE)-based analysis of over 27,000 older adults across 28 countries found that physical inactivity was the strongest lifestyle factor associated with severe pain, with odds nearly four times higher than those who regularly engage in moderate-energy activities (OR 4.35; 95% CI: 3.85–4.92) [43]. These findings parallel our exploratory results, which showed that frailty—a condition often marked by reduced physical activity—was significantly associated with higher pain levels. While our study was not designed to determine the directionality of this relationship, the frequent co-occurrence of pain and frailty highlights a need for further research. Interventions that incorporate both physical activity and multimodal pain management strategies may be particularly valuable in addressing the symptom burden and enhancing resilience in older adults with cancer.

### Clinical implications

These findings have important implications for both oncology and geriatric care. Routine assessment of patient-reported pain should be a standard component of oncology workflows, particularly at the initiation of chemotherapy. Early identification and management of uncontrolled pain may not only improve quality of life but also impact survival outcomes in older adults with cancer. Incorporating structured pain screening into comprehensive geriatric assessments can help identify high-risk individuals who may benefit from timely supportive care interventions. Enhancing access to multidisciplinary pain management—such as integrative medicine, cognitive behavioral therapy, and lifestyle-based approaches—may further improve symptom control and functional outcomes. Importantly, optimizing pain management could also help patients maintain chemotherapy intensity and reduce treatment delays. Encouraging even low-intensity physical activity may contribute to pain reduction and improved resilience, particularly in older adults with cancer [44].

### Limitations

This study, while providing valuable insights into the relationship between pain, survival, and frailty among older adults with newly diagnosed cancer, has several limitations that warrant consideration. The cohort is predominantly white and includes only three cancer types (lung, colorectal, and breast cancer), and only includes individuals who were treated with chemotherapy. As a result, the findings may not be generalizable to more diverse populations, individuals with other cancer types, or those managed without chemotherapy. Additionally, the cross-sectional nature of the pain assessment does not capture the dynamic nature of pain over the course of cancer treatment and progression. As a result, our findings may not fully reflect the impact of changes in pain intensity over time on overall survival. Addressing these limitations in future research through longitudinal studies, the inclusion of broader and more diverse populations, multidimensional pain and frailty assessments, and the consideration of a wider range of confounding factors will be crucial in further elucidating the complex interplay between pain, frailty, and survival in older adults with cancer. In addition, although vital status was primarily determined using the EMR, which may under-ascertain deaths, we also used the cancer registry to supplement mortality data. One study found that 19% of seriously ill primary care patients were not documented as deceased in the EMR alone [45]. However, since our analysis included both EMR and registry data—and all patients were monitored by the registry—we believe the risk of missing deaths in our cohort is likely lower.

## Conclusion

This study demonstrates that patient-reported pain is significantly associated with overall survival in older adults newly diagnosed with cancer, reinforcing its role as a prognostic indicator and potential target for intervention. The strength of this association highlights the need for improved pain assessment and management strategies in oncology care. Emerging evidence linking physical inactivity to severe pain further supports the potential role of movement-based interventions in symptom management. By addressing pain through targeted strategies—including lifestyle modifications and multimodal pain management—there is an opportunity to improve both quality of life and survival outcomes for older adults undergoing cancer treatment.

## Supplementary Information

Below is the link to the electronic supplementary material.

Supplementary file 1 (DOCX 14.8 KB)

## Data Availability

No datasets were generated or analysed during the current study.

## Declarations

### AI Usage Statement

ChatGPT was used to assist with grammar and proofreading during manuscript preparation. All content was reviewed and approved by the authors.
