# Supplementary material for: Patient-reported pain severity is associated with shorter survival in older adults with newly diagnosed cancer
Source: Support Care Cancer. 2025 Jul 26;33(8):722. doi: 10.1007/s00520-025-09779-x (PMC12296996; doi:10.1007/s00520-025-09779-x)
Supplement: Supplementary file 1 — (DOCX 14.8 KB) [file 520_2025_9779_MOESM1_ESM.docx]

| Supplement Table 1: Relationship Between Prior Opioid Use and Patient-Reported Pain Severity Before Chemotherapy Initiation | | | | |
| --- | --- | --- | --- | --- |
| Opioid Use | Pain Level (n,%) | | | |
|  | None | Mild | Moderate | Severe |
| No | 204 (87.9%) | 72 (85.7%) | 64 (64.0%) | 45 (51.1%) |
| Yes | 28 (12.1%) | 12 (14.3%) | 36 (36.0%) | 43 (48.9%) |

Footnote: Opioid use refers to documented opioid prescriptions prior to chemotherapy initiation. Pain levels were assessed using the Numeric Rating Scale (0–10) and categorized as none (0), mild (1–4), moderate (5–7), and severe (8–10). Percentages represent the proportion of patients within each pain category who did or did not use opioids prior to treatment.
